# Differential Hypermethylation of Death-Associated Protein Kinase Promoter in Central Neurocytoma and Oligodendroglioma

**DOI:** 10.1155/2014/506458

**Published:** 2014-04-27

**Authors:** Chia-Li Chung, Hung Pei Tsai, Cheng-Yu Tsai, Wan-Tzu Chen, Ann-Shung Lieu, Chih-Jen Wang, Jason Sheehan, Chee-Yin Chai, Aij-Lie Kwan

**Affiliations:** ^1^Department of Surgery, Kaohsiung Municipal Hsiao-Kang Hospital, Kaohsiung, Taiwan; ^2^Graduate Institute of Medicine, College of Medicine, Kaohsiung Medical University, Taiwan; ^3^Department of Neurosurgery, Kaohsiung Medical University Hospital, 100 Shih-Chuan First Road, Kaohsiung, Taiwan; ^4^Department of Pathology, Kaohsiung Medical University Hospital, Kaohsiung Medical University, Kaohsiung, Taiwan; ^5^Department of Surgery, Faculty of Medicine, College of Medicine, Kaohsiung Medical University, Kaohsiung, Taiwan; ^6^Department of Neurosurgery, University of Virginia, Charlottesville, VA, USA; ^7^Department of Pathology, College of Medicine, Kaohsiung Medical University, Kaohsiung, Taiwan

## Abstract

*Background*. Central neurocytoma and oligodendroglioma are rare tumors of the central nervous system. However, diagnosis between these two types of tumors is challenging due to their many cytological and histological similarities. Death-associated protein kinase (DAPK) is a calcium/calmodulin-regulated serine/threonine protein kinase involved in many apoptosis pathways, and repressed expression of DAPK by promoter hypermethylation has been found in a variety of human cancers. The purpose of this study was to assess DAPK protein expression and promoter hypermethylation in central neurocytoma and oligodendroglioma. *Method*. Central neurocytoma and oligodendroglioma samples were obtained from age- and sex-matched patients. DAPK protein expression was performed using immunohistochemical assays in formalin-fixed, paraffin-embedded sections. DAPK promoter hypermethylation was carried out using bisulfite-modified genomic DNA in methylation-specific PCR followed by separation in agarose gels. *Findings*. A statistically significant difference (*P* = 0.021) in DAPK promoter hypermethylation between central neurocytoma (76.9%) and oligodendroglioma (20%) was observed. High levels of DAPK protein expression were generally found in oligodendroglioma (90%), compared with 38.5% in central neurocytoma (*P* = 0.054; not statistically significant). There was an inverse correlation between DAPK protein expression and DAPK promoter hypermethylation in the cohort of 23 patients (*P* = 0.002). *Conclusions*. The results show that DAPK promoter hypermethylation and repressed expression of DAPK protein were more common in central neurocytoma than in oligodendroglioma. Thus, DAPK promoter hypermethylation could be useful for differential diagnosis between these two types of tumors, whereas DAPK protein expression might be less predictive. The role of DAPK promoter hypermethylation in the pathogenesis of central neurocytoma warrants further study.

## 1. Introduction

Central neurocytomas are rare tumors of the central nervous system, comprising only 0.1–0.5% of all brain neoplasms [1, 2]. Generally, central neurocytoma affects young adults with the tumors most frequently localizing in the supratentorial ventricular system and demonstrating calcification on computed tomography (CT) images [3, 4] although various cases of extraventricular neurocytoma have also been reported [5–9]. Despite a substantial advancement in the diagnosis and management [10–12] since its initial description reported in 1982 [13], central neurocytoma is still often confused with other tumors of the central nervous system, especially oligodendrogliomas.

Oligodendrogliomas occur primarily in the cortex and white mater of the cerebral hemispheres of adults in their fourth and fifth decades of life, while projection into the ventricles has also been found [14]. Histologically, central neurocytomas and oligodendrogliomas are characterized by sheets of monotonous cells with round nuclei surrounded by clear cytoplasm [15]. Thus, differentiation between oligodendroglioma and neurocytoma is challenging [16], and a definite diagnosis for these two types of tumors requires other complementary evaluations. Genetically, loss of heterozygosity on chromosomes 1p and 19q has been unequivocally found in the majority of oligodendroglioma patients [2, 17], whereas such codeletion in subjects with neurocytomas is still a matter of debate. Fujisawa et al. found no allelic loss on chromosomes 1p and 19q in central neurocytomas [17], while Rodriguez et al. and Tong et al. reported that 1p19q loss was seen in the majority of patients with extraventricular and central neurocytomas, respectively, although common regions of deletion could not be identified [18, 19]. A more convincing differentiation between central neurocytomas and oligodendrogliomas has been provided by immunohistochemical studies. Expression of Olig2 is seen in all oligodendrogliomas, whereas none or little expression of this transcription factor in central neurocytoma has been found [20, 21]. On the other hand, expression of the neuronal marker synaptophysin is observed in nearly all patients with central neurocytomas but rarely found in oligodendrogliomas [3, 11, 22]. Nevertheless, differentiation between central neurocytomas and oligodendrogliomas based on biochemical studies has not received much attention.

Oligodendroglioma cells can actively induce neuronal damage by releasing molecules able to inhibit neurite sprouting and to eventually cause apoptotic neuronal death [23, 24]. As for central neurocytoma, there is still no such published report aiming for the involving cell death pathway. The important roles of protein kinases in various cancers have long been recognized [25]. Death-associated protein kinase (DAPK) is a calcium/calmodulin-regulated serine/threonine protein kinase involved in many apoptotic pathways [26, 27]. Repressed expression of DAPK by promoter hypermethylation has been found in a variety of human cancers, such as colorectal carcinoma [28], soft tissue leiomyosarcoma [29], bladder cancer [30], and ulcerative colitis-associated carcinoma [31], to name a few. However, there has been no publication concerning the role of DAPK in central neurocytoma or oligodendroglioma. The purpose of this study was to assess DAPK protein expression and promoter hypermethylation in central neurocytoma and oligodendroglioma.

## 2. Materials and Methods

### 2.1. Patients

This study was approved by the Kaohsiung Medical University Hospital Review Board. Central neurocytomas and oligodendrogliomas were obtained from age- and sex-matched patients (ranged from 15 to 47 yr; 8 males and 5 females in each group) treated at the Kaohsiung Medical University Hospital. The specimens were diagnosed by H&E stain under light microscopy and immunostaining of synaptophysin and glial fibrillary acidic protein. All the 13 central neurocytomas are located intraventrically. All the 10 oligodendrogliomas are low grade tumors. The diagnosis was confirmed by physicians. Consents were received from all patients. Each tissue was divided into two equal parts, one for DNA extraction and the other for immunohistochemical staining.

### 2.2. DNA Extraction and Bisulfite Modification

Tissue samples from central neurocytoma and oligodendroglioma patients were digested with proteinase K at 56°C overnight, and genomic DNA was isolated by phenol-chloroform extraction using a commercially available kit according to the manufacturer's procedures. Approximately 2 *μ*g of tumor DNA was further modified by sodium bisulfite to convert unmethylated cytosines to uracils, and the modified DNA was eluted into buffer EB (Qiagen, Hilden, Germany). This bisulfite conversion and clean-up of genomic DNA were performed using the EpiTect Bisulfite kit (Qiagen). Purified DNA was used immediately as a template for methylation-specific polymerase chain reaction (PCR) described below or stored at −70°C until use.

### 2.3. Methylation-Specific PCR

Approximately 0.2 *μ*g of modified DNA was added to a PCR solution containing 1x PCR buffer, 1.25 mM MgCl_2_, 0.25 mM dNTP, 0.5 *μ*M PCR primers, and 1.25 U of GoTaq DNA polymerase (Invitrogen) in a total volume of 25 *μ*L. The forward and reverse primer sequences used for methylated DNA were 5′-GGATAGTCGGATCGAGTTAACGTC-3′ and 5′-CCCTCCCAAACGCCGA-3′, respectively, whereas the forward and reverse primer sequences used for unmethylated DNA were 5′-GGAGGATAGTTGGATTGAGTTAATGTT-3′ and 5′-CAAATCCCTCCCAAACACCAA-3′, respectively [32]. The CpGenome Universal Methylated DNA (Chemicon Int.) was used as positive control, and water was utilized as negative control. Amplification was carried out in a 2720 Thermal Cycler (ABI) at 95°C for 10 min followed by 35 cycles at 95°C for 4 s, 60°C for 60 s, and 72°C for 60 s. Afterwards, a 10 min extension was allowed at 72°C. The PCR products were then separated on 2% agarose gels and visualized after staining with ethidium bromide. Hypermethylation of DAPK genes was defined when DNA bands were detected in the agarose gel using PCR products generated from methylated primers or from both unmethylated and methylated primers. On the other hand, nonmethylation of DAPK genes was defined only when DNA bands were visible using PCR products obtained from unmethylated primers.

### 2.4. Immunohistochemical Staining

For immunohistochemical staining, tissues were fixed in formalin, embedded in paraffin, and cut into 5 *μ*m sections. They were then stained with hematoxylin and eosin and were evaluated to determine the extent of tumor cells presented in the sections using a light microscope. Subsequently, the samples were washed with PBS and incubated with anti-DAPK antibody (Santa Cruz) at a 1 : 100 dilution for 1 hr at room temperature. Afterwards, slides were washed for 30 min in PBS and incubated with secondary antibody (Dako Code K5007). Specimens were again washed with PBS, incubated with peroxidase-labeled streptavidin (DAB; Dako Code K3468) for measurement of the intensity of immunoreactivity.

Each section was given two independent scores, namely, the extent of tumor cells in the sample and the intensity of immunoreactivity, by an investigator blinded to the experiment. A score of 0 (zero) was assigned to a section if the extent of tumor cell was <1%, whereas scores of 1, 2, and 3 were given to sections with 1%–10%, 11%–50%, and >50% tumor cells, respectively. Likewise, a section received a score of 0 (zero) when the intensity of the slide was similar to the background level. Intensity scores of 1, 2, and 3 were assigned to sections with weak, moderate, and strong intensity of immunoreactivity, respectively. The values of these two independent parameters were multiplied to generate the final score for each section (ranging from 0 to 9) according to a published procedure [29]. A final score of <4 in a sample was considered as low DAPK expression, while a score of ≥4 was regarded as high DAPK expression.

### 2.5. Statistical Analysis

All results were expressed as mean ± SEM. An analysis of variance (ANOVA) followed by Fisher's exact test was performed to determine statistical significance between two groups. A *P* value of <0.05 was considered statistically significant.

## 3. Results

### 3.1. DAPK Promoter Hypermethylation

**Figure 1:**
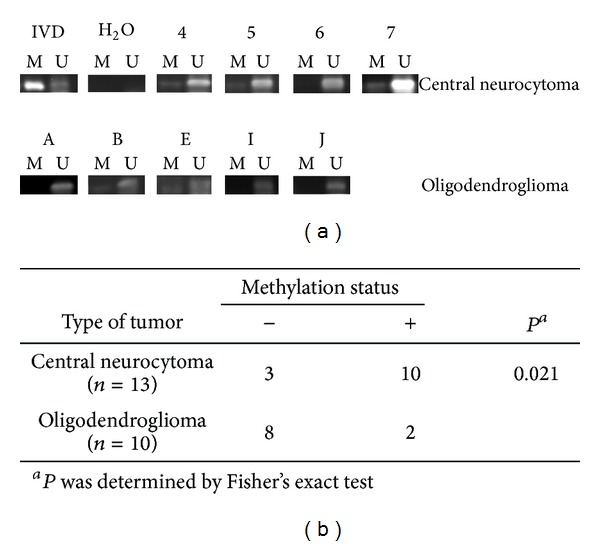
DAPK promoter hypermethylation in central neurocytoma and oligodendroglioma. Genomic DNA from patients with central neurocytoma or oligodendroglioma were extracted and analyzed for DAPK promoter hypermethylation as described in the methods. The upper panel shows representative results of the methylation-specific PCR products analyzed in agarose gels. IVD,* in vitro* methylated DNA used as positive control; H_2_O, negative control; M, methylated DAPK promoter gene; U, unmethylated DAPK promoter; and underlined numbers and letters, codes for patients. The methylation status of DAPK promoter in all patients is summarized in the lower panel. Eighty percent of central neurocytoma samples showed DAPK promoter hypermethylation. The result is statistically different from that in oligodendrogliomas, where only 20% of samples had methylated DAPK promoter.

DAPK promoter hypermethylation was observed in both central neurocytoma and oligodendroglioma (Figure 1(a)). Interestingly, 76.9% of central neurocytoma samples displayed DAPK promoter hypermethylation, while only 20% of oligodendrogliomas showed such an effect (Figure 1(b)). The difference was found to be statistically significant (*P* = 0.023).

### 3.2. DAPK Protein Expression

**Figure 2:**
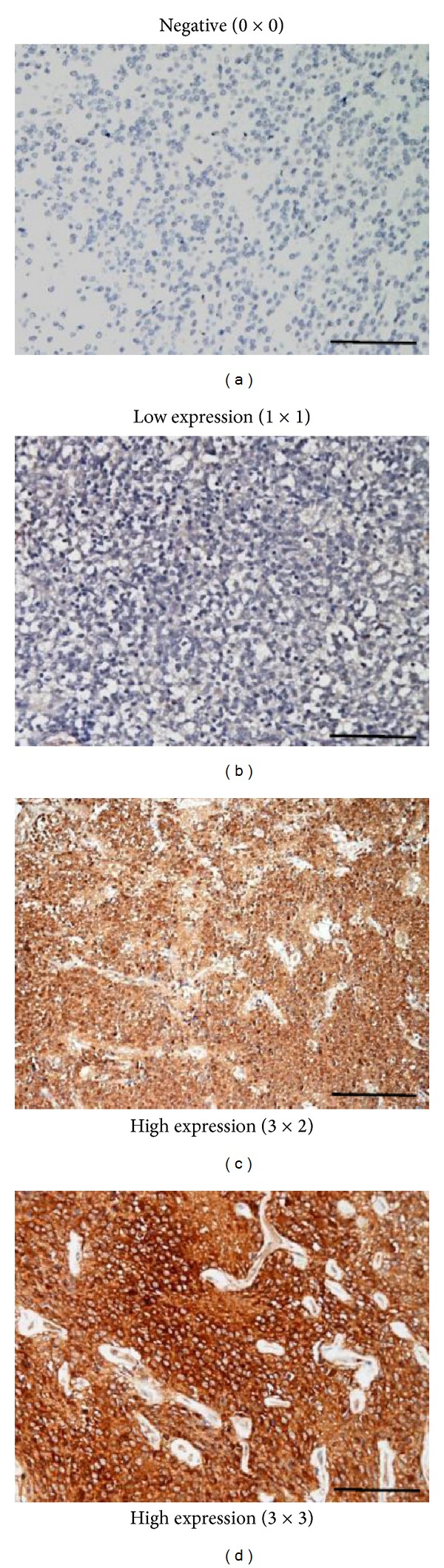
DAPK protein expression. The expression of DAPK protein in central neurocytoma and oligodendroglioma was assessed by both the extent of tumor cells and the intensity of immunoreactivity. These two parameters were graded numerically (0–3) as described in the methods. Representative slides from negative, low, and high DAPK protein expression are shown. Numbers (3 × 2, e.g.) in each parenthesis indicate assigned grade for extent of tumor cells × assigned grade for intensity of immunoreactivity. Scale bar, 100 *μ*m.

As mentioned in the methods, DAPK protein expression was assessed by both the extent of tumor cells and the intensity of immunoreactivity [29]. These two parameters were graded numerically (0–3), and their product was used to determine the level of protein expression. Representative slides from negative, low, and high DAPK protein expression are shown in Figure 2.  Table 1 summarizes the results of DAPK protein expression in patients with central neurocytoma and oligodendroglioma. A high level of DAPK protein expression was common in oligodendroglioma and was seen in 90% of patients. In contrast, only 38.5% of samples from central neurocytoma displayed high levels of DAPK protein expression. However, this difference observed between central neurocytoma and oligodendroglioma was not statistically significant (*P* = 0.054).

### 3.3. Correlation between DAPK Promoter Hypermethylation and Protein Expression

An effort was made to correlate DAPK promoter hypermethylation and DAPK protein expression in all of the central neurocytoma and oligodendroglioma samples evaluated. It was found that samples with low levels of DAPK protein expression always exhibited high levels of DAPK promoter hypermethylation. This was seen in 9 out of 9 cases (Table 2) from 1 oligodendroglioma and 8 central neurocytoma samples. In contrast, in the majority of samples with high levels of DAPK protein expression, unmethylated DAPK promoter was detected (11 out of 14 cases or 78.6% from 9 oligodendrogliomas and 5 central neurocytomas) (Table 2). These results showed that there was an inverse correlation between DAPK protein expression and DAPK promoter hypermethylation in the cohort of 23 patients (*P* = 0.002).

## 4. Discussion

Central neurocytoma and oligodendroglioma are rare tumors of the central nervous system. However, diagnosis between these two types of tumors is challenging due to their many cytological and histological similarities [15, 16, 33]. Subsequent genetic discoveries on the loss of heterozygosity in chromosomes 1p and 19q from the majority of oligodendroglioma patients [2, 17] as well as immunohistochemical studies showing differential expression of Olig2 [20, 21] and synaptophysin [3, 11, 22] in oligodendroglioma and central neurocytoma, respectively, have greatly helped the diagnosis. The most significant finding reported herein is the differentiation between central neurocytomas and oligodendrogliomas using biochemical methods. DAPK promoter hypermethylation was found in 80% of central neurocytomas but in only 20% of oligodendrogliomas (Figure 1). Although a high level of DAPK protein expression was common in oligodendroglioma (90%), this value is not significantly different from the 40% found in central neurocytoma. Thus, DAPK promoter hypermethylation could be useful for differential diagnosis between these two types of tumors, whereas DAPK protein expression might be less predictive.

Upon analysis of data obtained from both central neurocytoma and oligodendroglioma samples, there was an inverse correlation between DAPK protein expression and DAPK promoter hypermethylation (Table 2). These results are consistent with the findings from other studies showing DAPK promoter hypermethylation leads to a concomitant loss of DAPK protein expression in various cancers [26, 34]. Further analysis reveals that the repressed expression of DAPK protein derived mainly from central neurocytomas (8 out of 9 cases) (Table 1). The results suggest that DAPK promoter hypermethylation and repressed expression of DAPK protein are more common in central neurocytoma than in oligodendroglioma. It implies that DAPK promoter hypermethylation may play a role in the pathogenesis of central neurocytoma. Therefore, it is envisaged that agents capable of reversing this hypermethylation process may be novel drugs for the treatment of central neurocytoma.

Besides being localized centrally, neurocytomas have also been found extraventricularly [5–9]. It would be of interest to investigate whether or not DAPK promoter hypermethylation and reduced DAPK protein expression also apply to neurocytomas of these origins. A positive outcome would allow a more general statement on the differentiation between neurocytomas and oligodendrogliomas biochemically. Further studies are needed to clarify this matter.

## Figures and Tables

**Table 1:** Summary of the results of DAPK protein expression in central neurocytoma and oligodendroglioma.

Type of tumor	DAPK protein expression		*P*
	Low	High	
Central neurocytoma (*n* = 13)	8	5	0.054
Oligodendroglioma (*n* = 10)	1	9	

DAPK protein expression in the formalin-fixed, paraffin-embedded sections from central neurocytoma and oligodendroglioma was determined by two parameters, that is, the extent of tumor cells and the intensity of immunoreactivity. A numerical grade (0–3) from each parameter was assigned to every section, and the product of the two numbers was used to assess the level of DAPK protein expression as described in Section 2. A final score of <4 in a sample was considered as low DAPK expression, while a score of ≥4 was regarded as high DAPK expression. No statistically significant difference was found in the distribution of DAPK protein expression between central neurocytoma and oligodendroglioma.

**Table 2:** Correlation between DAPK protein expression and DAPK promoter hypermethylation in combined central neurocytoma and oligodendroglioma samples.

DAPK protein expression	Methylation status		*P*
	−	+	
Low (*n* = 9)	0	9	0.002
High (*n* = 14)	11	3	

DAPK protein expression and DAPK promotor hypermethylation were determined as described in Section 2. Results obtained from both the central neurocytoma and oligodendroglioma samples were combined in this analysis. In 9 out of 9 cases (100%), low levels of DAPK protein expression exhibited high levels of DAPK promoter hypermethylation. In contrast, in samples with high levels of DAPK protein expression, only 21.4% (3 out of 14 cases) showed high levels of DAPK promoter hypermethylation. These results showed the DAPK protein expression and DAPK promoter hypermethylation correlated inversely in the cohort of 23 patients (*P* = 0.002).

## References

[1] Bertalanffy A, Roessler K, Koperek O, Gelpi E, Prayer D, Knosp E (2005). Recurrent central neurocytomas. *Cancer*.

[2] Sharma MC, Deb P, Sharma S, Sarkar C (2006). Neurocytoma: a comprehensive review. *Neurosurgical Review*.

[3] Chen C-L, Shen C-C, Wang J, Lu C-H, Lee H-T (2008). Central neurocytoma: a clinical, radiological and pathological study of nine cases. *Clinical Neurology and Neurosurgery*.

[4] Schmidt MH, Gottfried ON, von Koch CS, Chang SM, McDermott MW (2004). Central neurocytoma: a review. *Journal of Neuro-Oncology*.

[5] Agarwal S, Sharma MC, Sarkar C (2011). Extraventricular neurocytomas: a morphological and histogenetic consideration. A study of six cases. *Pathology*.

[6] Chou S, Varikatt W, Dexter M, Ng T (2010). Extraventricular neurocytoma with atypical features and ganglionic differentiation. *Journal of Clinical Neuroscience*.

[7] Cook DJ, Christie SD, Macaulay RJB, Rheaume DE, Holness RO (2004). Fourth ventricular neurocytoma: case report and review of the literature. *Canadian Journal of Neurological Sciences*.

[8] Gomes FL, França LR, Zymberg ST, Cavalheiro S (2006). Central neurocytomas of uncommon locations: report of two cases. *Arquivos de Neuro-Psiquiatria*.

[9] Yang G-F, Wu S-Y, Zhang L-J, Lu G-M, Tian W, Shah K (2009). Imaging findings of extraventricular neurocytoma: report of 3 cases and review of the literature. *American Journal of Neuroradiology*.

[10] Amini E, Roffidal T, Lee A (2008). Central neurocytoma responsive to topotecan, ifosfamide, carboplatin. *Pediatric Blood and Cancer*.

[11] Jaiswal S, Vij M, Rajput D (2011). A clinicopathological, immunohistochemical and neuroradiological study of eight patients with central neurocytoma. *Journal of Clinical Neuroscience*.

[12] Terakawa Y, Tsuruno T, Ishibashi K, Okada Y, Shimotake K, Murata T (2010). Central neurocytoma presenting with massive hemorrhage leading to coma—case report. *Neurologia Medico-Chirurgica*.

[13] Hassoun J, Gambarelli D, Grisoli F (1982). Central neurocytoma. An electron-microscopic study of two cases. *Acta Neuropathologica*.

[14] Ludwig CL, Smith MT, Godfrey AD, Armbrustmacher VW (1986). A clinicopathological study of 323 patients with oligodendrogliomas. *Annals of Neurology*.

[15] Klysik M, Gavito J, Boman D, Miranda RN, Hanbali F, De Las Casas LE (2010). Intraoperative imprint cytology of central neurocytoma: the great oligodendroglioma mimicker. *Diagnostic Cytopathology*.

[16] Makuria AT, Henderson FC, Rushing EJ, Hartmann D-P, Azumi N, Ozdemirli M (2007). Oligodendroglioma with neurocytic differentiation versus atypical extraventricular neurocytoma: a case report of unusual pathologic findings of a spinal cord tumor. *Journal of Neuro-Oncology*.

[17] Fujisawa H, Marukawa K, Hasegawa M (2002). Genetic differences between neurocytoma and dysembryoplastic neuroepithelial tumor and oligodendroglial tumors. *Journal of Neurosurgery*.

[18] Rodriguez FJ, Mota RA, Scheithauer BW (2009). Interphase cytogenetics for 1p19q and t(1;19)(q10;p10) may distinguish prognostically relevant subgroups in extraventricular neurocytoma. *Brain Pathology*.

[19] Tong CYK, Ng H-K, Pang JCS, Hu J, Hui ABY, Poon W-S (2000). Central neurocytomas are genetically distinct from oligodendrogliomas and neuroblastomas. *Histopathology*.

[20] Okada M, Yano H, Hirose Y (2011). Olig2 is useful in the differential diagnosis of oligodendrogliomas and extraventricular neurocytomas. *Brain Tumor Pathology*.

[21] Preusser M, Budka H, Rössler K, Hainfellner JA (2007). OLIG2 is a useful immunohistochemical marker in differential diagnosis of clear cell primary CNS neoplasms. *Histopathology*.

[22] Wharton SB, Chan KK, Hamilton FA, Anderson JR (1998). Expression of neuronal markers in oligodendrogliomas: an immunohistochemical study. *Neuropathology and Applied Neurobiology*.

[23] D’Agostino S, Salamone M, di Liegro I, Vittorelli ML (2006). Membrane vesicles shed by oligodendroglioma cells induce neuronal apoptosis. *International Journal of Oncology*.

[24] Lo Cicero A, Schiera G, Proia P (2011). Oligodendroglioma cells shed microvesicles which contain TRAIL as well as molecular chaperones and induce cell death in astrocytes. *International Journal of Oncology*.

[25] Brognard J, Hunter T (2011). Protein kinase signaling networks in cancer. *Current Opinion in Genetics and Development*.

[26] Michie AM, McCaig AM, Nakagawa R, Vukovic M (2010). Death-associated protein kinase (DAPK) and signal transduction: regulation in cancer. *The FEBS Journal*.

[27] Schumacher AM, Velentza AV, Watterson DM (2002). Death associated protein kinase as a potential therapeutic target. *Expert Opinion on Therapeutic Targets*.

[28] Mittag F, Kuester D, Vieth M (2006). DAPK promotor methylation is an early event in colorectal carcinogenesis. *Cancer Letters*.

[29] Kawaguchi K-I, Oda Y, Saito T (2004). Death-associated protein kinase (DAP kinase) alteration in soft tissue leiomyosarcoma: promoter methylation or homozygous deletion is associated with a loss of DAP kinase expression. *Human Pathology*.

[30] Tada Y, Wada M, Taguchi K-I (2002). The association of Death-associated Protein Kinase hypermethylation with early recurrence in superficial bladder cancers. *Cancer Research*.

[31] Kuester D, Guenther T, Biesold S (2010). Aberrant methylation of DAPK in long-standing ulcerative colitis and ulcerative colitis-associated carcinoma. *Pathology Research and Practice*.

[32] Esteller M, Sanchez-Cespedes M, Resell R, Sidransky D, Baylin SB, Herman JG (1999). Detection of aberrant promoter hypermethylation of tumor suppressor genes in serum DNA from non-small cell lung cancer patients. *Cancer Research*.

[33] Mut M, Güler-Tezel G, Lopes MBS, Bilginer B, Ziyal I, Özcan OE (2005). Challenging diagnosis: oligodendroglioma versus extraventricular neurocytoma. *Clinical Neuropathology*.

[34] Gozuacik D, Kimchi A (2006). DAPk protein family and cancer. *Autophagy*.

